# The role of age and AMH on cumulative live birth rates over multiple frozen-thawed embryo transfer cycles: a study based on low prognosis patients of POSEIDON 3 and 4 groups

**DOI:** 10.1186/s12958-024-01243-5

**Published:** 2024-06-17

**Authors:** Lan Xia, Xiaowei Zhou, Xiaoling Wang, Shen Zhao, Xian Wu, Huihui Xu, Aijun Zhang, Zhihong Niu

**Affiliations:** Department of Obstetrics and Gynecology, Ruijin Hospital, Shanghai Jiao Tong University School of Medicine, Shanghai, China

**Keywords:** Cumulative live birth, POSEIDON criteria, Low prognosis, Age, anti-müllerian hormone

## Abstract

**Background:**

Among the POSEIDON criteria, group 3 and group 4 have an expected low prognosis. For those patients with inadequate ovary reserve, embryo accumulated from consecutive oocyte retrieval cycles for multiple frozen-thawed embryo transfers (FET) has become more common. It is necessary to inform them of the pregnancy outcomes after single or multiple FET cycles before the treatment. However few studies about cumulative live birth rate (CLBR) for those with low prognosis have been reported.

**Methods:**

This retrospective study included 4712 patients undergoing frozen embryo transfer cycles from July 2015 to August 2020. Patients were stratified as POSEIDON group 3, group 4, control 1 group (< 35 years) and control 2 group (≥ 35 years). The primary outcome is CLBRs up to six FET cycles and the secondary outcomes were LBRs per transfer cycle. Optimistic approach was used for the analysis of CLBRs and the depiction of cumulative incidence curves.

**Results:**

Under optimistic model analyses, control 1 group exhibited the highest CLBR (93.98%, 95%CI 91.63-95.67%) within 6 FET cycles, followed by the CLBR from women in POSEIDON group 3(92.51%, 95%CI 77.1-97.55)was slightly lower than that in control 1 group. The CLBR of POSEIDON group 4(55% ,95%CI 39.34-70.66%)was the lowest and significantly lower than that of control 2 group(88.7%, 95%CI 80.68-96.72%). Further, patients in POSEIDON group 4 reached a CLBR plateau after 5 FET cycles.

**Conclusions:**

The patients of POSEIDON group 3 may not be considered as traditional “low prognosis” in clinical practice as extending the number of FET cycles up to 6 can archive considerably CLBR as control women. While for the POSEIDON group 4, a simple repeat of the FET cycle is not recommended after four failed FET cycles, some strategies such as PGT-A may be beneficial.

## Introduction

Since the delivery of the first IVF baby nearly 40 years ago, great steps forward have been made in assisted reproductive technology (ART). However, the live birth rates (LBR) per treatment cycle have stagnated over the past couple of decades at around 26–32% [1]. Generally, multiple cycles are required to achieve a live birth, especially in women with low prognosis. Compared with LBR per cycle, cumulative live birth rate (CLBR) of consecutive cycles could provide a more accurate and individualized information for clinicians and couples [2], thus, CLBR has been used by national registries in many countries, such as Australia, Germany, the United Kingdom, and the United States [3].

The emergence of POSEIDON criteria (Patient-Oriented Strategies Encompassing IndividualizeD Oocyte Number) grouped patients based on both oocyte quality (age) and number of oocytes (ovarian reserve) [4] helps specialists stratify the low prognosis patients more effectively from the perspective of treatment. Among the criteria, POSEIDON group 3 and group 4 indicated those with inadequate ovary reserve (AMH<1.2ng/ml or AFC<5) which predicted poor response prior of ovary stimulation. It has been reported that the LBR of Poseidon group 3 and group 4 in a single fresh embryo transfer cycle are lower than that of the control groups [5]. However, to our knowledge, since POSEIDON criteria were introduced within the last decade, the estimating of CLBR based on multiple frozen embryo transfer (FET) cycles in low prognosis women is rarely explored.

Our study, therefore, aimed to quantify cumulative live birth rates over consecutive frozen embryo transfer cycles in low prognosis women belonging to POSEIDON group 3 and group 4 and provide new individualized treatment recommendations, especially after recurrent implantation failures.

## Materials and methods

### Study participants

We conducted a retrospective cohort study on patients undergoing frozen-thawed embryo transfer from July 2015 to August 2020 at the reproductive center of the Ruijin Hospital affiliated with the medical school of Shanghai Jiaotong University. All included patients were between the age of 22 and 45 years, had a body mass index (BMI) of > 18 kg/m^2^ and < 35 kg/m^2^, and a morphologically normal uterus on salpingogram and/or hysteroscopy. Patients undergoing egg donation or preimplantation genetic testing (PGT) were excluded from the study. In addition, women with fresh embryo transfer at least once or endometrium thickness < 7 mm after estrogenization in FET cycle were excluded. All enrolled data were anonymous and from those women who allowed the use for retrospective studies with written informed consent. The research was approved by the reproductive ethics committee of Ruijin Hospital.

**Fig. 1:**
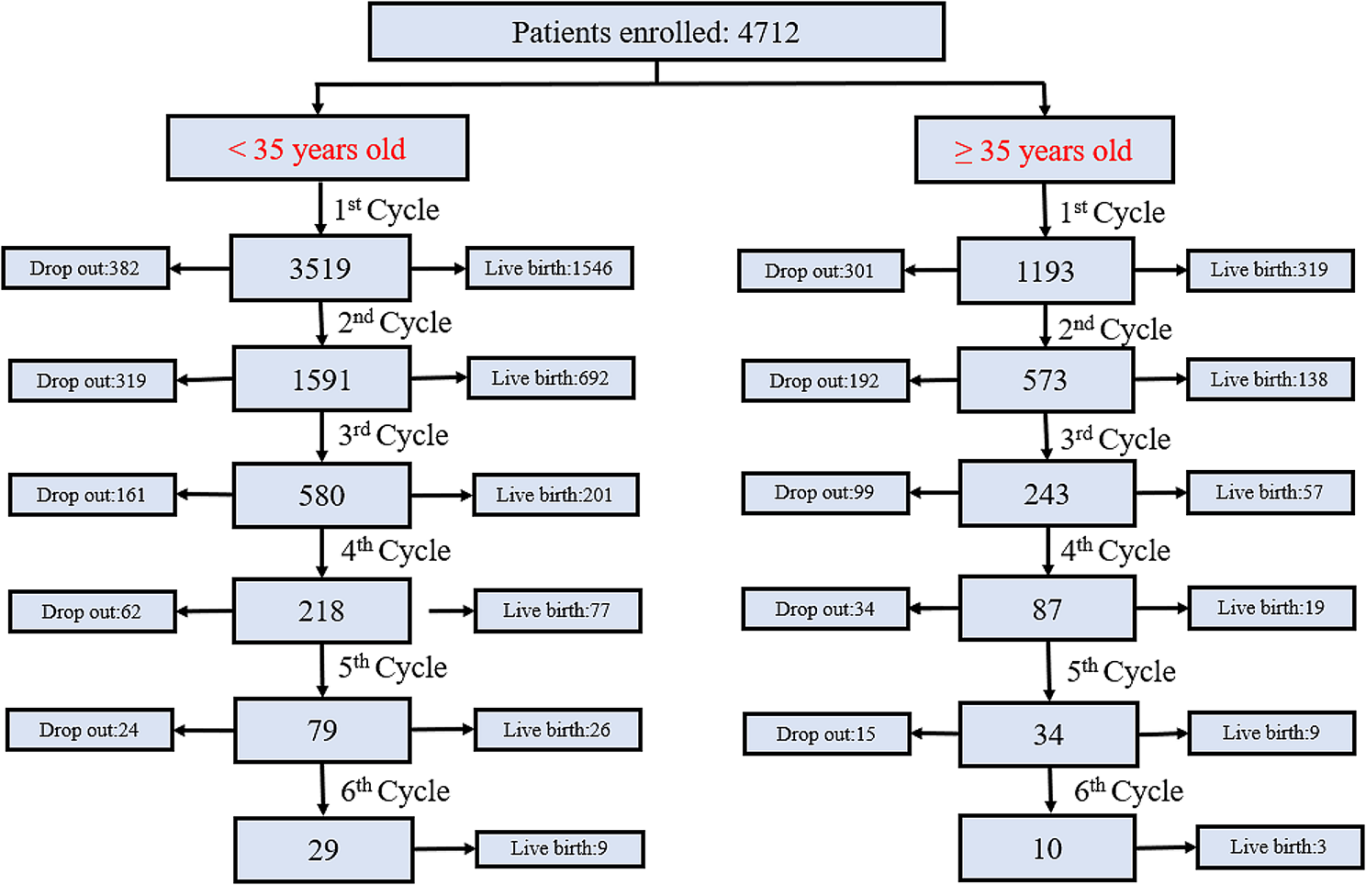
Study flow chart

To investigate the impact of the decreased ovarian reserve on CLBRs in young and advanced age women, we defined women with a normal ovarian reserve (AFC ≥ 5 and AMH ≥ 1.2ng/ml) as control groups. Control 1 group involved women with age < 35 years, and control 2 group involved women of ≥ 35 years old. Figure 1 displayed the flow chart of the study. We calculated the CLBR up to six cycles in the analysis as too few patients underwent a seventh cycle. For women who stopped treatment before achieving a LB would be considered as drop-out.

### Treatment regimen

IVF treatment was monitored and managed according to the standardized clinical protocols as previously reported [6]. Briefly, ovarian stimulation was performed with human menopausal gonadotropin (HMG) and recombinant follicle-stimulating hormone (r-FSH). Pituitary inhibition was obtained by gonadotropin-releasing hormone (GnRH) agonist or GnRH antagonist. Oocyte retrieval was performed transvaginally under ultrasound guidance at 34–36 h after trigger. The oocytes were fertilized by traditional in vitro fertilization (IVF) or intra cytoplasmic sperm injection (ICSI). After 3 to 6 days of culture, the freeze-all embryo strategy was performed for all included patients.

The transfer of embryos was managed as our previously report [6]. The endometrial was prepared with hormone replacement treatment (HRT). Briefly, Estradiol (E2) valerate (Progynova®; Schering, Germany) was commenced orally on the 2nd or 3rd day of a natural or progesterone-induced menstrual cycle, and 10–12 days later, ultrasound examination was carried out to measure endometrial thickness. When the endometrial thickness attained ≥ 7 mm, progesterone vaginal gel (Crinone®, Merck Serono, Switzerland) was administered at a dose of 90 mg/d. Cleavage-stage embryos or blastocysts were transferred 3 or 5 days after progesterone supplementation in hormone replacement treatment cycle. Warming protocols were conducted following traditional methods according to the instructions of the Vit Kit (Kitazato Biopharma, Japan). Embryos were brought progressively back to 37 °C and cultured for 2 h in the cleavage medium and the blastocyst medium after the warming procedure and before transfer.

### Outcome measures

We defined live birth as an infant born showing any sign of life after 28 weeks gestation. The primary outcome of this study is CLBRs which was defined as the probability of at least one live birth resulted from one or multiple frozen embryo transfer cycles. The secondary outcomes were LBRs per transfer cycle.

### Statistical analysis

The data were analyzed using the Statistical Package for Social Sciences (SPSS, SPSS Inc., Chicago, IL, USA), MedCalc statistical software (version 12.7.5; http://www.medcalc.org), and SAS (version 9.4, SAS Institute Inc., Cary, NC). The continuous data are presented as mean ± SD. Analysis of variance test was used to compare mean values. The categorical data are represented as frequencies (percentage). Chi-squared test and Fisher’s exact test were used to compare rates among groups. The Kaplan–Meier method was used to report CLBR. *P* < 0.05 was considered as statistically significant. The hazard ratio for live birth in the analysis of overall survival was estimated with the use of a stratified Cox proportional-hazards model. The sample size is calculated using Medcalc statistical software. It is found [7] that the cumulative pregnancy rate of Poseidon 3 and 4 groups is about 35% and 10% respectively after the third ET cycle. An a priori analysis for proportions using the difference from the constant model was performed, leading to a suggested sample size of 172 and 176.

## Results

### Baseline and ART characteristics

A total of 4712 women were involved in the study. They underwent 8156 embryo transfer cycles during the study period, which resulted in 3096 deliveries.

The baseline characteristics of all the cohorts are summarized in Table 1. When compared with control group, the mean number of oocytes retrieved, cryopreserved embryos per cycle and proportion of cycles with oocytes retrieved, embryos cryopreserved was significantly lower and the numbers of oocyte retrieval cycles was significantly higher in POSEIDON group 3 and POSEIDON group 4 respectively. Besides, there was significantly lower proportion of blastocyst transferred in POSEIDON group 3 and 4 compared to the control group.

The results presented in Table 2 provide a comprehensive overview of the perinatal and neonatal outcomes of the four groups.

**Table 1:** Patient demographics and treatment cycle characteristics

			<35							≥ 35			
		Control 1 group		POSEIDON group 3			*P*-value		Control 2 group		POSEIDON group 4		*P*-value
Patient		3182		337					795		398		
Age		30.42 ± 2.92		29.75 ± 2.71		0.082			37.95 ± 2.46		38.91 ± 2.65	0.065	
BMI (kg/m2)		23.03 ± 3.30		22.76 ± 3.16		0.192			23.1 ± 3.24		23.07 ± 3.22	0.880	
Infertility etiology (%)													
Male factor(%)		682(21.43)		69(20.47)		0.683			182(22.89)		84(21.1)	0.272	
Tubal factor		2112(66.37)		218(64.69)		0.336			504(63.39)		253(63.57)	0.954	
Endometriosis		192(6.03)		21(6.23)		0.495			49(6.16)		26(6.53)	0.928	
Ovulation disorders		54(1.70)		9(2.67)		0.199			14(1.76)		9(2.26)	0.998	
Unexplained		142(4.47)		20(5.93)		0.090			46(5.80)		26(6.53)	0.112	
Type of infertility (%)						0.248						0.161	
Primary		2203(69.23)		223(66.17)					354(44.53)		159(39.95)		
Secondary		979(30.77)		114(33.83)					441(55.47)		239(60.05)		
Cumulative numbers of oocyte retrieval cycles		1.32 ± 0.72		2.18 ± 1.55		<0.01			1.71 ± 1.22		2.83 ± 2.03	<0.01	
Cycles with oocytes retrieved (%, *n*)		99.7(4372/4385)		96.19 (706/734)		<0.01			99.28 (1385/1395)		93.75 (1065/1136)	<0.01	
NO. of oocytes retrieved		12.84 ± 5.54		5.00 ± 3.86		<0.01			8.65 ± 4.97		3.28 ± 2.83	<0.01	
Fertilization rate		78.61(44,240/56,276)		77.91 (2864/3676)		0.31			78.79(9508/12,067)		78.54 (2927/3727)	0.74	
Cycles with embryos cryopreserved (%, *n*)		96.62 (4237/4385)		80.79 (593/734)	<0.01				89.82 (1253/1395)		71.83 (816/1136)	<0.01	
Cryopreserved embryos per cycle		4.99 ± 3.32		1.98 ± 1.83	<0.01				3.26 ± 2.57		1.36 ± 1.34	<0.01	
Stage of embryo transferred					<0.01							<0.01	
Cleavage (%, *n*)		55.35 (3025/5464)		64.67 (357/552)					65.65 (973/1482)		75.08 (494/658)		
Blastocyst (%, *n*)		44.64 (2439/5464)		35.33 (195/552)					34.34 (509/1482)		24.92 (164/658)		
Number of embryos transferred per cycle		1.61 ± 0.24		1.56 ± 0.25	0.104				1.56 ± 0.25		1.54 ± 0.25	0.52	

*Note* BMI = Body Mass Index; Fertilization rate = The Number Of 2pn Oocytes /The Number of Total Oocytes

**Table 2:** Perinatal and neonatal outcomes

			<35						≥ 35			
		Control 1 group		POSEIDON group 3		*P*-value		Control 2 group		POSEIDON group 4		*P*-value
Miscarriage rate (%, *n*)		16.80 (483/2875)		16.87 (41/243)	0.98			25.47 (149/585)		30.94 (56/181)	0.15	
Ectopic pregnancy		1.36 (39/2875)		1.65 (4/243)	0.71			1.71(10/585)		3.31(6/181)	0.18	
Gestational diabetes (%, *n*)		5.61(132/2353)		5.05(10/198)	0.74			7.51(32/426)		8.40(10/119)	0.75	
Gestational hypertension (%, *n*)		3.14(74/2353)		3.54(7/198)	0.76			4.69(20/426)		5.88(7/119)	0.60	
Postpartum hemorrhage (%, *n*)		0.72(17/2353)		0.51(1/198)	0.73			0.94(4/426)		0.84(1/119)	0.92	
Preterm birth (< 37 weeks) (%, *n*)		4.12(97/2353)		4.04(8/198)	0.96			4.46(19/426)		5.04(6/119)	0.80	
Low birthweight (< 2500 g)(%, *n*)		4.16(98/2353)		4.55(9/198)	0.80			4.69(20/426)		5.88(7/119)	0.60	
High birthweight (> 4000 g) (%, *n*)		6.97(164/2353)		7.58(15/198)	0.75			7.28(31/426)		8.4(10/119)	0.68	

### Live birth rate according to age or the POSEIDON criteria

**Fig. 2:**
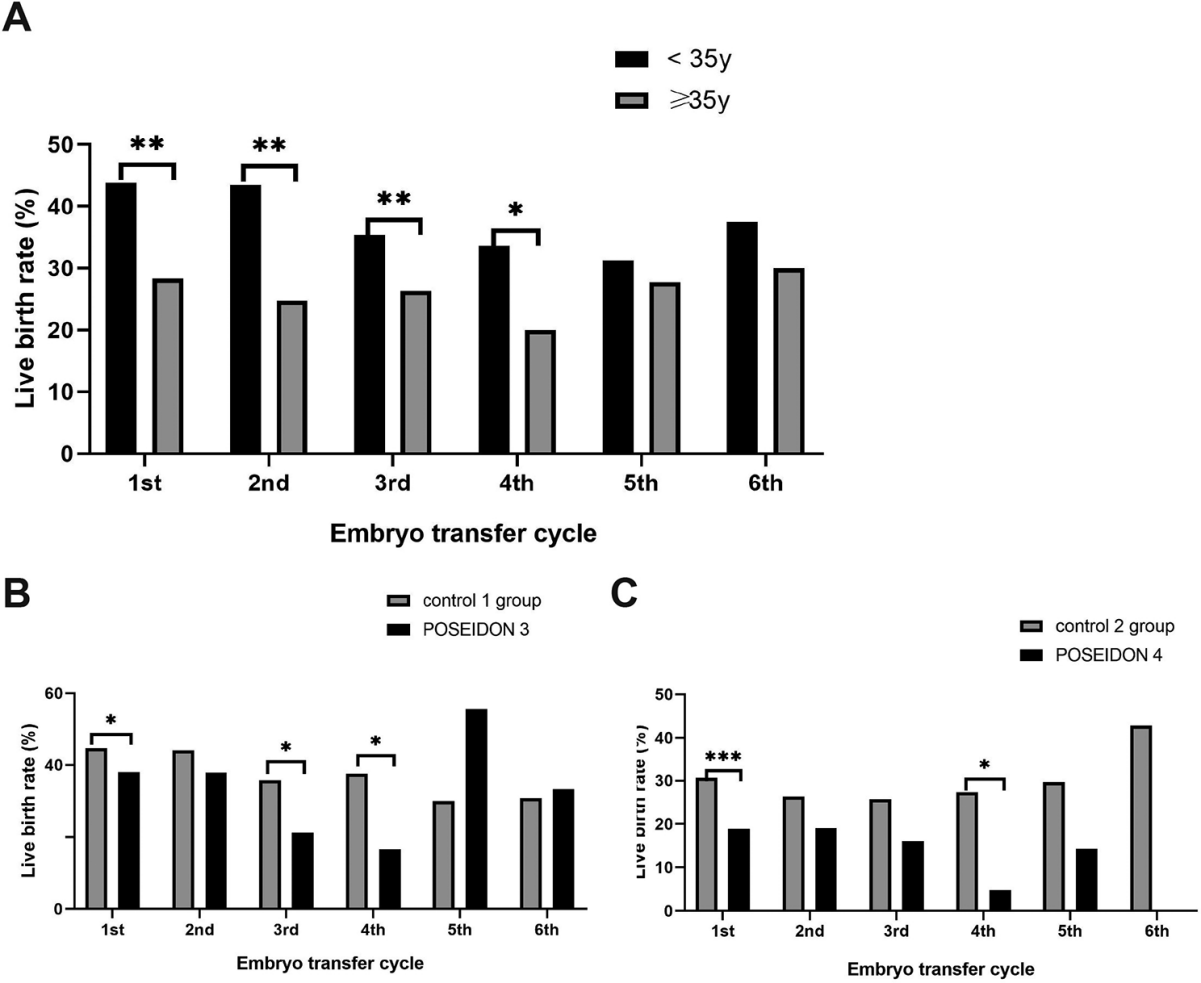
The LBRs per ET cycle (**A**) comparisons based on age (≥ 35 years and < 35 years old). (**B**) comparisons between POSEIDON group 3 and control 1 group. (**C**) comparisons between POSEIDON group 4 and control 2 group

The LBR per cycle according to maternal age through the six cycles are shown in Fig. 2A. During the 1st to the 4th ET cycle, the women aged ≥ 35 years had significantly decreased LBRs compared with younger women(26.74% V.S. 43.84%, 24.77% V.S. 43.47%, 26.37% V.S. 35.38%, 20% V.S.33.65%, ,respectively, *P*<0.05). However, the LBR in the 5th and the 6th ET cycle between the two group did not differ significantly (27.78% & 31.25% and 30% & 37.5%).

The live birth rate according to POSEIDON criteria through the six ET cycles are shown in Fig. 2B and C. The LBR in the 1st, 3rd, 4th in the POSEIDON group 3 were significantly lower when compared to the control 1 group (37.98% V.S. 44.56%, 21.28% V.S.35.83%, 16.67% V.S.37.63%, *P*<0.05). While the LBR in POSEIDON group 4 differed significantly only in 1st and 4th FET cycle when compared to the control 2 group (30.69% V.S.18.84%, 27.27% V.S. 4.76%, *P*<0.05 ).

### CLBR according to age

**Fig. 3:**
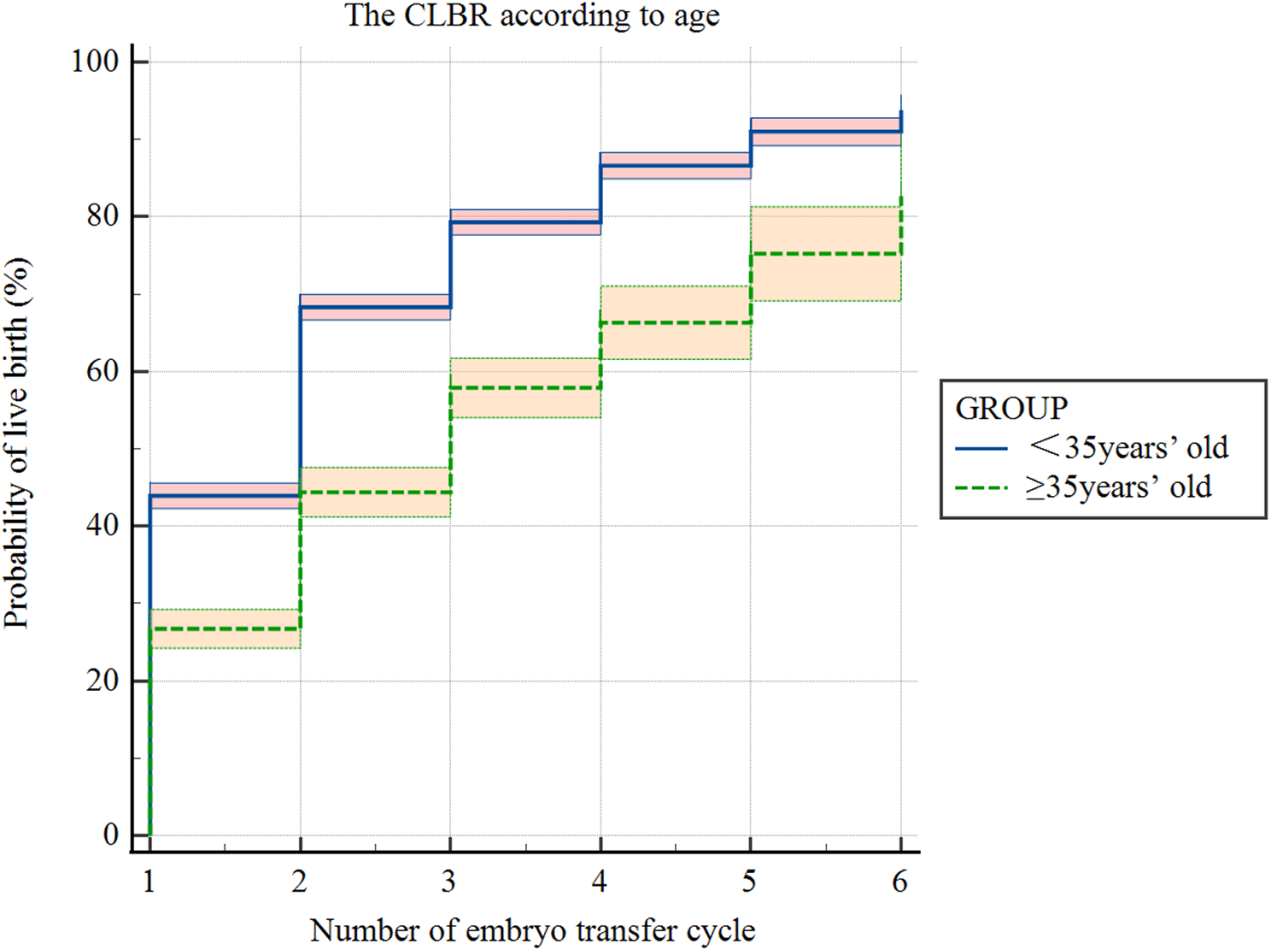
The CLBR according to age using a standard Kaplan Meier survival model. The colored band surrounding the Kaplan Meier curve represents the 95% CI

The cumulative live birth rates after up to 6 ET cycles grouped by age are shown in Table 3; Fig. 3. The overall CLBR was 91.6%(95%CI 89.4-93.8%)as illustrated by Kaplan-Meier estimates reporting. The CLBR was 82.7% (95%CI 74.47-90.93%)for women ≥ 35 years and 93.8% (95%CI 91.85-95.75%)for women aged < 35 years. The log-rank test revealed a significant difference in CLBR across age subgroups (*P* < 0.0001).

**Table 3:** The CLBRs according to maternal age

FET cycle	Cumulative birth rate(95%CI)			*P*-value	Overall
	<35years		≥ 35years		
1	43.9(42.26–45.54)	26.7(24.19–29.21)		< 0.0001	39.6(38.2–41)
2	68.3(66.65–69.95)	44.4(41.21–47.59)		< 0.0001	62.8(61.29–64.31)
3	79.3(77.67–80.93)	57.9(54.04–61.76)		< 0.0001	74.5(72.93–76.07)
4	86.6(84.91–88.29)	66.3(61.58–71.02)		< 0.0001	82.4(80.69–84.11)
5	91.02(89.23–92.81)	75.2(69.1–81.3)		< 0.0001	87.9(85.99–89.81)
6	93.8(91.85–95.75)	82.7(74.47–90.93)		< 0.0001	91.6(89.4–93.8)

CI = Confidence Interval

### CLBR according to POSEIDON criteria

**Fig. 4:**
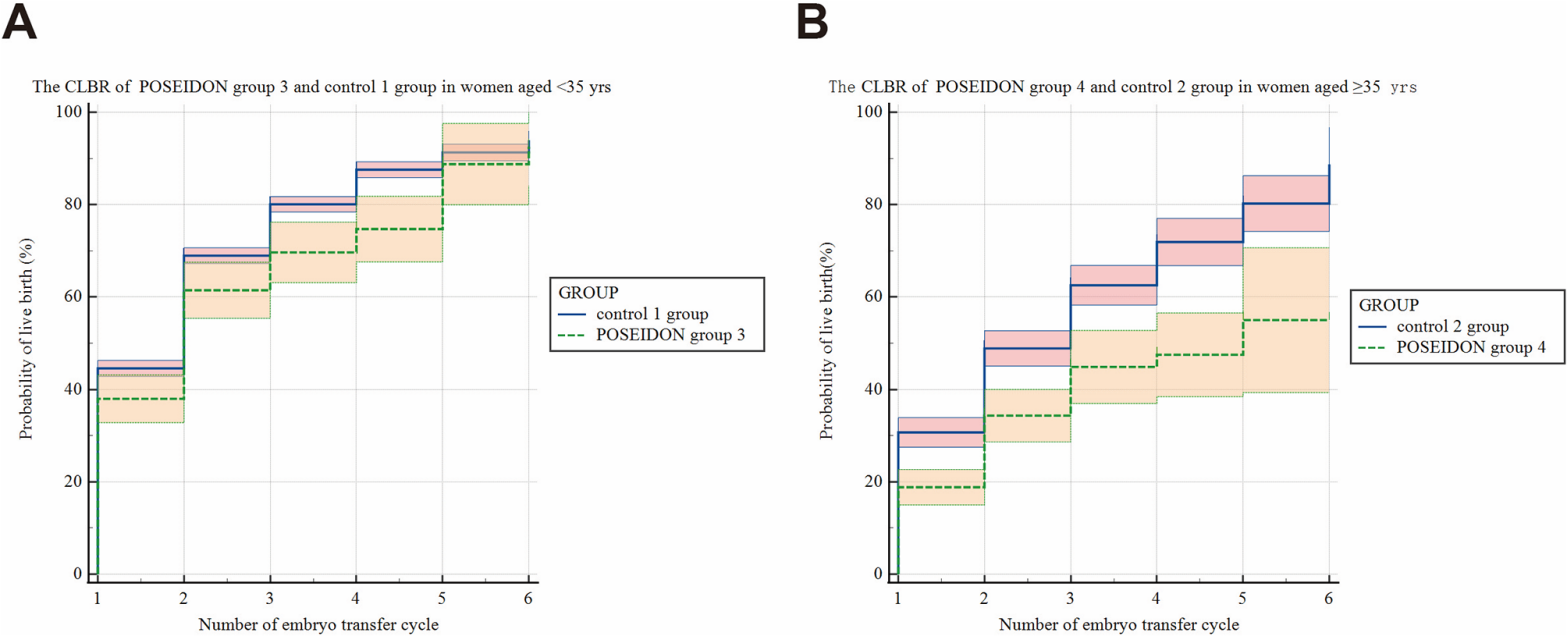
The CLBR according to POSEIDON Criteria using a standard Kaplan Meier survival model. (**A**) The CLBR in POSEIDON 3 and control 1 group. (**B**) The CLBR in POSEIDON 4 and control 2 group. The colored band surrounding the Kaplan Meier curve represents the 95% CI

The cumulative live birth rates calculated according to POSEIDON criteria were shown in Table 4; Fig. 4 illustrated by Kaplan-Meier estimates reporting. In women < 35 years old, the CLBR is 92.51% (95%CI 77.1-97.55% )for POSEIDON group 3 and 93.98% (95%CI 91.63–95.67%)for control 1 group. In women aged ≥ 35 years, the CLBR is 55% (95%CI 39.34-70.66%)for POSEIDON group 4 and 88.7% (95%CI 80.68-96.72%)for control 2 group. The log-rank test revealed a significant difference in CLBR across POSEIDON subgroups (*P* < 0.0001).

**Table 4:** The CLBRs according to POSEIDON criteria

Number of embryo transfers cycle	<35					≥ 35				
	Control 1 group	POSEIDON group 3	*P*-value		Control 2 group		POSEIDON group 4	*P*-value		
1	44.6 (42.87–46.33)	38 (32.83–43.17)	0.021		30.7 (27.49–33.91)		18.8 (14.96–22.64)	< 0.0001		
2	69 (67.29–70.71)	61.5 (55.44–67.56)	0.0074		48.9 (45.1–52.7)		34.3 (28.62–39.98)	< 0.0001		
3	80.1 (78.43–81.77)	69.7 (63.13–76.27)	0.0016		62.6 (58.33–66.87)		44.9 (36.98–52.82)	< 0.0001		
4	87.6 (85.89–89.31)	74.7 (67.6–81.8)	0.0005		71.9 (66.79–77.01)		47.5 (38.44–56.56)	< 0.0001		
5	91.3 (89.51–93.09)	88.8 (80.02–97.58)	0.001		80.2 (74.18–86.22)		55 (39.34–70.06)	< 0.0001		
6	93.98 (91.63–95.67)	92.51 (77.1–97.55)	0.0011		88.7 (80.68–96.72)		55 (39.34–70.06)	< 0.0001		

CI = Confidence Interval

### COX regression analysis

The association between probability of live birth for POSEIDON group 3 and 4 by cox regression model is presented in Table 5.

The prognosis according to the POSEIDON criteria was independent predictors of CLBR after adjustment for the stage of embryo transferred, and the aHR of POSEIDON group 3 was 0.835 (95%CI:0.722–0.962, *P* = 0.015) while the aHR of POSEIDON group 4 was 0.628 (95%CI: 0.512–0.77, *P* < 0.001) compared to the control group of the same age respectively.

**Table 5:** Cox regression analysis based on the POSEIDON criteria in the same age of CLBR factors

Risk factors	HR (95%CI)	*P* value		Adjust HR^*^ (95%CI)	*P* value
Control 1 group	Reference			Reference	
POSEDON group 3	0.832(0.72–0.962)		0.013	0.835(0.722–0.962)	0.015
Control 2 group	Reference			Reference	
POSEDON group 4	0.621(0.507–0.761)		< 0.001	0.628(0.512–0.77)	< 0.001

HR = Hazard Ratio; Ci = Confidence Interval

*Adjusted for the stage of embryo transferred

## Discussion

Low prognosis patients of POSEIDON groups 3 and 4 had significantly lower LBR and CLBR in multiple embryo transfer cycles [5]. However, almost all published studies being focused on CLBR of low prognosis women included both fresh and frozen embryo transfer cycles. Here, we performed the cohort study to tell the discrepancy of CLBR up to 6 cycles of frozen embryo transfer in women of POSEIDON groups 3 and 4, which is of great significance to clinical counseling before IVF as the increased utility of freeze-all strategy in modern ART practice. In our study, although the LBR of POSEIDON group 3 was lower in the first four ET cycles, while its CLBR could more or less “catch up” with the control group 1 after six ET cycles, which means that patients < 35 years with low AMH can benefit from the multiple ET cycles. However, for patients ≥ 35 years old, the disparity of the CLBR between POSEIDON group 4 and control 2 group increased with the number of FET cycles, and the last two cycles made little contribution to the CLBR for POSEIDON group 4.

### Interpretation of findings

Generally, the age-related reduction of LBR is mainly caused by the decreased oocyte quality. Higher embryo aneuploidy rates in women over 35 years old resulted in a corresponding decrease in implantation rate and the increase in miscarriage rate [8]. However, for those women undergoing implantation failure in consecutive four FET cycles, age did not make means in LBR in the following 2 ET cycles, which suggested that uterine factors, including uterine local immune environment, may contribute more for repeated implantation failure (RIF) in younger women. The study of Chen et al. [9] can support the perspective as they found premature aging of the endometrium exists in young women (<35 years old) with recurrent implantation failure. Another evidence came from the data that Preimplantation Genetic Testing for Aneuploidy (PGT-A) was proved to be effect in improving LBR in advanced age patients with RIF, but not in young RIF women [10].

In fresh IVF cycle, serum AMH was a predictor for LBR, which was mainly contributed by greater oocyte yield [11]. However, the role of serum AMH on LBR in frozen embryo transfer cycle was rarely studied. According to Li et al. [12], the LBR in the first FET cycle after oocyte retrieval was positively associated with AMH. and the reason may be higher chances of good quality embryo being selectable for transfer in the first cycle. Here, the comparison of LBR between POSEIDON group 3 or group 4 and control groups also suggested that LBR was higher in the first FET cycle for those with higher AMH, but not in all FET cycles.

Based on previous study, AMH and age were both independent predictors for CLBR [13]. Aimed to exclude the interference of fresh cycles, we only enrolled FET cycles following “freeze all” cycles in the study and observed the effects of AMH and age on CLBR. From the first to the sixth FET cycle, the CLBR of POSEIDON group 3 kept rising and the disparity of CLBR between POSEIDON group 3 and control 1 group was stepdown. Finally, the CLBR of two group of women was very closed. However, the CLBR disparity between POSEIDON groups 4 and control 2 group was increased over the course of six FET treatments. The CLBR of POSEIDON group 4 was finally much lower than control 2 group and reached plateau after 5 FET cycles Thus, the negative effect of AMH on CLBR is compensated by repeated cycles in young women but augmented in advanced age of women. It should be noticed that the CLBR of control 2 group reached 88% up to six FET cycles which suggested higher AMH could compensated the adverse impact of age to a certain extent in women older than 35 years. The mechanisms may be more oocytes retrieved resulted in more chances to select euploid embryos with good morphology to transfer.

The COX regression analysis confirmed that in FET cycles, POSEIDON criteria was an independent factor positively associated with CLBR. Until now, only one large cohort study on CLBR in FET cycles was published which analyzed the CLBR within 5 years or 9 FET cycles in groups based on POSEIDON criteria [14]. The CLBR over 5 years estimated by optimistic analytical method was 0.75 (0.71–0.78) in POSEIDON group 3, almost the same as that of control group with 0.79 (0.78–0.80) and much higher than 0.41 (0.37–0.46) in POSEIDON group 4. Interestingly, although they included up to 9 cycles, all patients reached a CLBR plateau after 3.5 years or 6 FET cycles. According to the published data and our results, although women of both POSEIDON group 3 and group 4 have diminished ovarian reserve, the noticeable differences in CLBR after 6 cycles of FET between the two groups indicated female age has more significant impact on the CLBR than the ovary reserve parameter. These results were in line with Hu’s study [13]. which reported that the age did not negative affect CLBR in those ≤ 35 years, while in 36–38 years, 39–40 years and 41–42 years old group, the adjusted HR of age on cumulative live birth is 0.71 (0.58–0.88), 0.45 (0.35–0.60) and 0.27 (0.19–0.38) for respectively. The higher embryos aneuploid rate was still the greatest factor affecting pregnant outcome in advanced age of women. Luo’s [15] report confirmed that in POSEIDON 4 group undergoing PGT-A, 61.7% women failed to obtain euploid embryos, while in group of POSEIDON 3 the rate was only 18%.

This study has some limitations. The first was the percentage of the patients who dropped out, which was also a common problem while researching cumulative outcomes. The reason for discontinuation of treatment may be physical and emotional strain, financial burden, or the lack of informative censoring from doctors. To account for this, we calculated ‘optimal’ estimates, which assumes that the cumulative live-birth rate in women who discontinue IVF had the equal chances of getting live birth to those who continue further treatments. Second, this was a single-center retrospective study and the retrospective nature of the study cannot exclude all biases resulted by uneven patients’ basic data, such as obesity, endometriosis, adenomyosis and so on. Future prospective studies in different. populations may be necessary to validate the results.

### Clinical implications

The reporting of the CLBR over multiple frozen embryo transfer cycles of low prognosis patients has important clinical implications. First, we were delighted to find out that young women with low ovary reserve could benefit from extending the number of ET cycles. So, for POSEIDON group 3 patients, more efforts should be focused on increasing the number of oocytes retrieved through tailored controlled-ovarian-stimulation (COS). Therefore, novel COS strategies, such as oocyte/embryo accumulation in consecutive cycles [16] or double ovarian stimulation in the same ovarian cycle [17], have been proposed. Also, as the oocyte quantitative parameters had limited predicting value in CLBR for women younger than 35 years, those women with lower AMH may not be considered as traditional “low prognosis” in clinical practice, or they should be informed more about optimistic outcomes after consecutive ET cycles than a single cycle.

Second, for POSEIDON group 4, as the increased rate of aneuploid embryos resulted higher risk of implantation failure and miscarriages, more oocyte yield may be not enough to compensate the decrease in oocyte quality. Thus, moreover, consultation should be conducted to inform the reduction of the probability of live birth and preimplantation genetic testing for aneuploidies (PGT-A) should be recommended after 3–4 cycles of implantation failure.

## Conclusion

In conclusion, the current study suggested age is the primary decisive factor for CLBR and ovarian reserve has limited value in predicting CLBR, especially in young women. Our findings support the efficacy of extending the number of ET cycles up to 6 to low prognosis patients of POSEIDON group 3, while for the POSEIDON group 4 PGT-A is strongly recommended after four FET cycles. The results of our study can help clinicians provide accurate, individualized counselling to help patients build realistic expectations for their reproductive outcomes, preparing emotionally and financially for their IVF journey.

## Data Availability

The data and materials supporting the findings of this study are available upon reasonable request. Researchers interested in accessing the data or materials should contact Lan Xia at xl40710@rjh.com.cn.
